# The Prevalence and Diagnostic of Silent Ischemic Heart Disease in Polish Kidney Transplant Candidates

**DOI:** 10.3390/jcm15124596

**Published:** 2026-06-13

**Authors:** Piotr B. Kuczera, Aleksandra Grzmil, Szymon Domagała, Jakub Milczarek, Anna Walukiewicz, Andrzej Więcek, Aureliusz Kolonko

**Affiliations:** 1 Student Scientific Society, Department of Nephrology, Transplantation and Internal Medicine, 40-027 Katowice, Poland; pkuczera@o2.pl (P.B.K.); o.grzmil@gmail.com (A.G.); szymon2768.92@wp.pl (S.D.); 2 Doctoral School of Medicine, Medical University of Silesia in Katowice, 40-055 Katowice, Poland; jakubmilczarek95@wp.pl; 3 Independent Public Teaching Hospital in Katowice, Francuska 20/24, 40-027 Katowice, Poland; annawalukiewicz@op.pl; 4 Department of Nephrology, Transplantation and Internal Medicine, Medical University of Silesia in Katowice, 40-027 Katowice, Poland; awiecek@sum.edu.pl

**Keywords:** atherosclerosis, cardiological assessment, coronary angiography, ischemic heart disease, kidney transplantation

## Abstract

**Background/Objectives**: Patients with chronic kidney disease (CKD) have an increased risk of ischemic heart disease (IHD). Some discrepancies exist between cardiological and nephrological guidelines regarding the extent of diagnostic procedures in CKD patients who are candidates for kidney transplantation. The aim of this study was to assess the cardiac status of these patients after cardiological checkup. **Methods**: The present study included all kidney transplant candidates referred to the Regional Qualification Center between January 2021 and February 2024. We characterized the group of patients in whom IHD was diagnosed during the cardiological checkup. **Results**: Among 346 patients, IHD was newly identified in 44 (12.7%) subjects. These patients were significantly older [median 62.9 (51.9–65.4) vs. 47.2 (36.8–57.9) years; *p* < 0.001], had longer dialysis vintage [median 20 (12.5–42) vs. 14 (6–31) months; *p* < 0.05] and were more frequently diabetic (29.6 vs. 16.9%, *p* < 0.05) than the rest of the study cohort. Of note, they were also characterized by significantly more frequent manifestation of atherosclerosis lesions visualized using routine imaging methods (i.e., chest X-ray and abdominal aorta and iliac artery visualization). The stepwise logistic regression analysis revealed that age [OR 1.05 (1.02–1.09); *p* <0.01] and the ad hoc atherosclerotic score [OR 1.88 (1.27–2.77); *p* < 0.001] independently predicted the diagnosis of IHD during the cardiological qualification of potential kidney transplant candidates. **Conclusions**: During the cardiological examination, IHD was diagnosed in a substantial number of kidney transplant candidates. The presence of atherosclerotic lesions detected by routine noninvasive vascular system imaging methods may suggest the need for extending IHD diagnostics even in relatively young patients without clinical symptoms.

## 1. Introduction

In patients with chronic kidney disease (CKD), the risk of cardiovascular complications, including ischemic heart disease (IHD), is significantly higher compared with individuals with normal kidney function, even after adjusting for confounding factors such as age and gender [1,2]. The long-lasting uremic milieu, including hypertension, dyslipidemia, uremic toxins, hypervolemia and persistent calcium-phosphate metabolism disturbances, especially worsened in dialysis patients, leads to accelerated atherosclerosis and arteriosclerosis [3,4,5,6]. Diabetes mellitus is an additional independent risk factor for vascular calcification [3,7]. Thus, to avoid major cardiovascular complications during and after kidney transplantation, careful cardiological screening is required before patient assignment to the active waiting list.

According to the current guidelines of the Polish Organizational and Coordination Center for Transplantation “Poltransplant”, routine pre-transplant cardiological assessment should be based on the analysis of electrocardiograms and echocardiography, followed by exercise stress tests, dobutamine tests, coronary calcium scores, scintigraphy and/or coronary angiography, if applicable (in patients >60 years of age or diabetics, those with ischemic heart disease or the signs of atherosclerosis) [8]. However, there are substantial discrepancies between cardiological and nephrological guidelines regarding the need for extensive diagnostic procedures in kidney transplant candidates. Moreover, the prognostic value of different methods of cardiac evaluation for post-transplant cardiovascular complications is still a matter of debate [4,9,10], especially in asymptomatic kidney transplant candidates [11,12,13]. As a result, during the primary assessment of kidney transplant candidates, especially those who are older and have long dialysis vintage, there are many doubts as to which patient really deserves extended cardiological diagnostics, despite a cardiologist’s optimistic opinion. It should also be mentioned that the diagnosis of coronary heart disease in a potential kidney transplant candidate provides an opportunity to implement appropriate treatment, which itself may reduce the risk of severe cardiovascular complications after transplantation. At this clinical stage, we look for potential confounders which may guide our decision concerning the need for additional cardiovascular imaging.

We are not aware of any previous study analyzing the association between the presence of vascular system atherosclerosis visualized by simple imaging methods and the risk of IHD diagnosis in kidney transplant candidates. Thus, the aim of the current study was to characterize patients with CKD stage 5 who were potential candidates for kidney transplantation with newly diagnosed IHD during the cardiological checkup, with special emphasis on who should be referred for extended cardiovascular screening during the pre-transplantation evaluation.

## 2. Materials and Methods

### 2.1. Study Group

This retrospective analysis consists of all 346 patients who were consecutively referred for qualification before assignment to the kidney transplantation waiting list between January 2021 and February 2024 at a single kidney transplant center in Katowice. Their demographic data and history of kidney disease, including renal replacement therapy modality to date as well as comorbidity, were considered. All analyzed data were obtained from the kidney transplant candidates’ qualification cards, sent electronically from the primary nephrological care center (dialysis unit, nephrology ward or an out-patient department) to the kidney transplant coordination center.

According to the opinion of the Bioethics Committee of the Medical University of Silesia (BNW/NWN/0052/KB/88/26), the present analysis based on anonymous patient data was permitted without obtaining individual informed consent. The study protocol fulfilled the Protocol of Helsinki criteria. The detailed course of general and cardiological evaluation of each transplant candidate was analyzed, including an anamnesis regarding previous cardiovascular diseases and complications, the primary cardiological assessment (including electrocardiogram (ECG) and echocardiography), the results of routine imaging tests (chest X-ray, abdominal ultrasound, USG-Doppler of iliac arteries (all of the above are obligatory) and carotid Doppler ultrasound (if available)), and the results of additional functional or imaging methods performed according to the suggestion of the cardiologist or, after the first review, by the transplant center coordinator. We identified and characterized the group of patients in whom IHD was found for the first time during the overall course of pre-transplant cardiological evaluation. IHD was diagnosed based on anatomic criteria, with both significant obstructive (≥50% stenosis of the left main coronary artery or ≥70% stenosis of any other major epicardial artery) and non-obstructive (if plaque is present but causes <50% stenosis) variants included in the present analysis.

The results of different imaging methods, including chest X-ray and ultrasound abdominal aorta and iliac artery visualization, were semi-quantitatively scored as positive or negative for signs of main vessel atherosclerosis. Based on these three single scores, the atherosclerosis score was calculated with a range of 0–3, depending on the number of analyzed points at which signs of atherosclerosis were present.

### 2.2. Statistical Analysis

Statistical analyses were performed using Statistica 13.3 PL for Windows (Tibco Inc., Palo Alto, CA, USA) and MedCalc v20.014 (MedCalc Software, Mariakerke, Belgium). Values were presented as means with 95% confidence interval, medians with interquartile ranges or frequencies. For comparisons between groups, Student’s *t*-test (for quantitative variables) or the χ^2^ test (for qualitative variables) was used. Variables with non-normal distribution were compared using the Mann–Whitney U-test. The Kruskal–Wallis test was used for comparisons between groups of patients allocated based on the number of atherosclerotic lesions detected in imaging examinations. Stepwise logistic regression analysis was performed with new diagnosis of IHD as the dependent variable and patient age, the occurrence of diabetes mellitus, dialysis vintage and the value of the atherosclerosis score as potential independent variables. The potential independent variables were selected based on the differences between the group of patients with new IHD and the rest of the cohort. For all analyses, a *p* value below 0.05 was considered statistically significant. Effect size was calculated according to the formula Z (module)/√n for the Mann–Whitney U test and the Cramer V test for the χ^2^ test.

## 3. Results

The mean age of the analyzed cohort was 48.4 (47.0–49.9) years, with 62.7% of the patients being males. The clinical characteristics are given in Table 1.

At the time of primary transplant referral, IHD was diagnosed in 42 subjects, and intervention was required in 23 patients. During the evaluation process, in 44 (12.7%) patients a new diagnosis of IHD was made, based on coronary angiography in 90.9% and other imaging methods (coronary scintigraphy or computed tomography of heart vessels) in 9.1% of patients.

Importantly, in 68.2% of evaluated patients, the IHD diagnosis was made during the primary cardiology screening, but in the remaining 31.8% of patients, additional coronary imaging was performed following the suggestion of the regional transplant qualification center. Six patients underwent one or more coronary artery stents, and one patient required coronary artery bypass grafting (CABG). The majority of patients with newly diagnosed IHD were referred for conservative treatment, including antiplatelet drugs and lipid-lowering therapy. They were also referred for further regular checkups with a cardiologist.

Patients in whom IHD was diagnosed during the kidney transplant evaluation process were significantly older than the rest of the candidates [median 62.9 (51.9–65.4) vs. 47.2 (36.8–57.9) years; *p* < 0.001; r = 0.31]. However, their age range was 40–73 years, with 9 (20.5%) between 40 and 50 years and the next 10 (22.7%) between 50 and 57 years. They were also characterized by considerably longer dialysis vintage [median 20 (12.5–42) vs. 14 (6–31) months; *p* = 0.013; r = 0.13] and were more frequently diabetic (29.6 vs. 16.9%, *p* = 0.044; V = 0.15). Twenty-seven (61.4%) patients were dialyzed using an arterio-venous fistula, 11 (25%) using a permanent catheter, 3 (6.8%) were treated with peritoneal dialysis and 3 (6.8%) were considered in the preemptive protocol. Three patients were waiting for their second kidney transplant.

**Figure 1:**
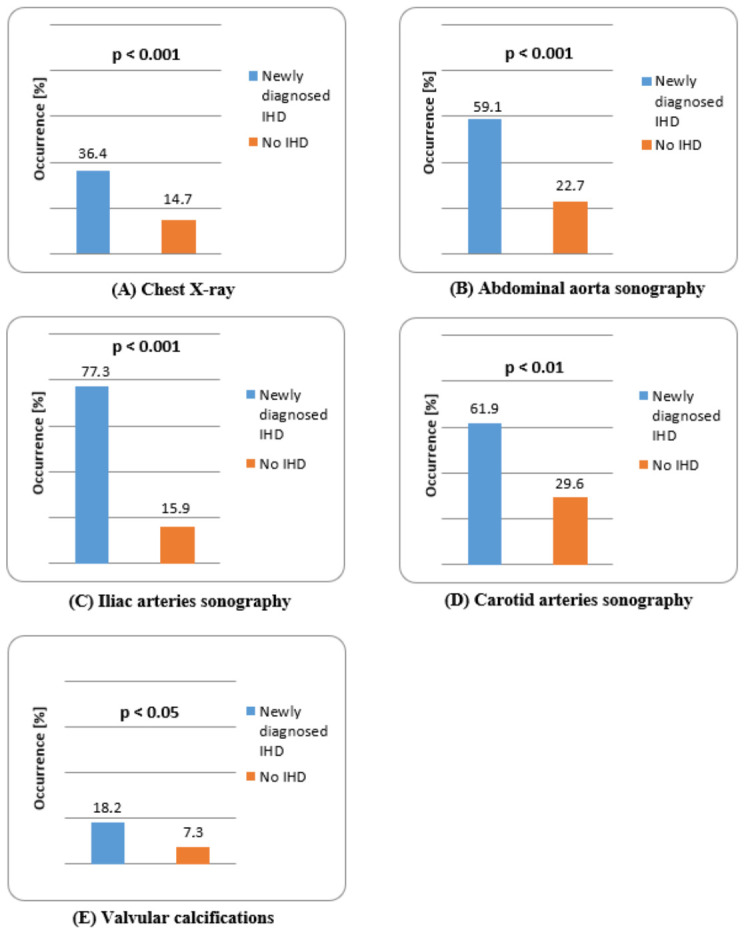
Comparison of the prevalence of atherosclerotic lesions detected by routine imaging methods in patients with newly diagnosed ischemic heart disease (IHD) and those without IHD. (**A**) Chest X-ray. (**B**) Abdominal aorta sonography. (**C**) Iliac arteries sonography. (**D**) Carotid arteries sonography. (**E**) Valvular calcifications.

Of note, this group of patients, when compared to the IHD-free subjects, was also characterized by significantly more frequent surrogate signs of atherosclerosis detected using routine imaging methods, including chest X-ray (36.4 vs. 14.7%; *p* < 0.001; V = 0.21), aorta assessment in an abdominal ultrasound (59.1 vs. 22.7%; *p* < 0.001; V = 0.29), iliac artery assessment in M-mode examination (77.3 vs. 15.9%; *p* < 0.001; V = 0.51), carotid artery ultrasound assessment (61.9 vs. 29.6%; *p* = 0.008; V = 0.26) and valvular calcifications in echocardiography (18.2 vs. 7.3%; *p* = 0.019; V = 0.14). The distribution of atherosclerotic lesions detected by routine imaging methods in patients with newly detected IHD and those without IHD is presented in Figure 1A–E.

**Figure 2:**
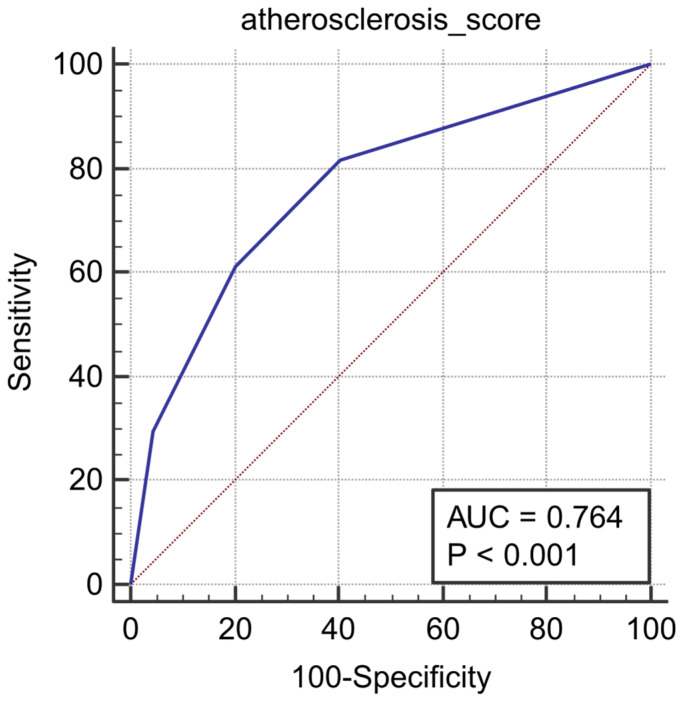
The ROC analysis for the atherosclerosis score to predict new IHD diagnosis.

Based on the atherosclerosis score calculated ad hoc from the results of all three obligatory imaging methods (chest X-ray and abdominal aorta and iliac artery visualization), these signs of distal atherosclerosis were noted in 0, 1, 2 or 3 different locations in 18.2, 20.5, 31.8 and 29.5%, respectively. As expected, the number of imaging test results presenting atherosclerotic lesions significantly increased along with increasing patient age (χ^2^ = 8.66; *p* < 0.05). The ROC analysis revealed that atherosclerosis score >1 predicted the presence of newly diagnosed IHD with 79.8% specificity and 61.4% sensitivity (Figure 2).

The stepwise logistic regression analysis revealed that age [OR 1.05 (1.02–1.09); *p* < 0.01] and the ad hoc atherosclerotic score [OR 1.88 (1.27–2.77); *p* < 0.001] independently predicted a diagnosis of IHD during the cardiological qualification of potential kidney transplant candidates [χ^2^ = 48.24, *p* < 0.0001, AUC 0.812, 95% CI 0.765–0.853)]. Neither the length of dialysis therapy nor the presence of diabetes mellitus was included in the final model.

In the follow-up period of 21 months, 38 (86.4%) patients with IHD diagnosed during the referral for the waiting list and 263 (87.1%) from the rest of the study cohort were successfully transplanted in a median time from their addition to the waiting list of 113 (IQR, 25–362) vs. 202 (IQR, 80–428) days (*p* = 0.033, R = 0.08).

## 4. Discussion

The long-lasting discussion concerning the effectiveness and utility *versus* the risk and costs of pre-transplant cardiac evaluation has focused primarily on the prognostic value of such a screening in regard to cardiovascular complications or mortality after transplantation. Alongside patients with already known history of IHD or previous cardiovascular complications, the pre-transplant cardiac screening should identify those asymptomatic CKD patients with significant vascular pathology in whom interventional treatment would allow further transfer to the active waiting list [14]. While there is no doubt of the value of coronary angiography in high-risk patients [15,16], most of the recent commentaries underline the lack of evidence-based guidelines in this field [13,14] and even advocate for the complete abolition of cardiac screening in asymptomatic patients [14,17], as it generates costs, increases the risk of post-procedure complications and may delay the patient’s active listing [17]. In fact, even among those screened, only a minority required revascularization [11,18], and the occurrence of post-transplant MACE was low [12,18]. However, in a recent metanalysis, the mean prevalence of American Heart Association class 1 indications for revascularization ranged from 0% to 17%, but 10% of patients had multivessel coronary artery disease and 35% were referred for invasive angiography [19].

Nevertheless, the population of CKD stage 5 patients is specific, with a very high burden of cardiovascular complications [20] and several non-traditional risk factors [5,21]; thus, general cardiology guidelines may not be fully applicable in kidney transplant candidates [22]. It should be stressed that a substantial percentage of asymptomatic dialysis patients presents severe coronary stenosis [9]. Additionally, contrary to the general population, the performance of exercise electrocardiography for the identification of IHD is worse than that of resting ECG due to the preexisting ECG abnormalities [23]. As a consequence, this specific population of patients requires very accurate and often beyond-the-guidelines pre-transplant cardiologic evaluation to increase the chance of reliable IHD diagnosis. This hypothesis was partly confirmed by our present study, as the number of asymptomatic patients diagnosed with IHD only during the extended cardiological assessment was slightly larger than the number of patients with IHD diagnosed based on clinical signs or symptoms and routine diagnostics prior to transplant referral.

Coronary computed tomography angiography (CTA) was described as a suboptimal technique due to its modest specificity (66%) [9]; however, three out of four studies analyzed in this meta-analysis had a high risk of patient selection bias [24]. Moreover, the analyzed studies were performed more than a decade ago, and since then, technical CT equipment performance and radiological interpretation accuracy have markedly improved. Myocardial perfusion scintigraphy by single-photon emission CT (SPECT) is another diagnostic option for IHD, which was demonstrated to be the most cost-effective in comparison with PET and CTA [25]. Finally, dobutamine stress echocardiography was shown to have a sensitivity of 0.79 and a specificity of 0.89 in detecting coronary artery stenosis in kidney transplant candidates [26]. Thus, while invasive coronary angiography is reserved for symptomatic CKD patients, the abovementioned imaging or functional tests should be more broadly considered during careful qualification of potential kidney transplant candidates.

It is worth noticing that finding IHD during the pre-transplant screening process offers a chance for adequate interventional or non-interventional treatment, whose aim is both to decrease the risk of post-transplant MACE and to improve long-term patient survival, independently of transplantation. Moreover, patients diagnosed with IHD may benefit from referral for regular cardiological checkup, both before and after transplantation. However, in the current literature there is still a lack of practical hints addressing the dilemma of which asymptomatic CKD stage 5 patients should undergo in-depth diagnostics for IHD.

In the present study, we identified and characterized a group of CKD patients referred for kidney transplantation, in whom a first diagnosis of IHD was made based on detailed cardiological evaluation. Of note, it was shown that the decision concerning a more invasive diagnostic approach may be effectively supported by the presence of atherosclerotic lesions in routinely available examinations of vascular imaging, including chest X-ray, aorta and iliac artery USG-Doppler, and carotid artery visualization, with the special clinical usefulness of a simply calculated atherosclerosis score. These imaging results are usually routinely performed in potential transplant candidates and should be available during the first transplant referral. Such a strategy may generally shorten the time of pre-transplant checkup and successful active listing. Although only 15.9% of those subjects finally underwent coronary stenting or CABG, in a substantial percentage of the remained patients, conservative treatment with lipid-lowering and antiplatelet drugs was started thereafter, which may be longitudinally beneficial for dialysis or transplanted CKD patients [27].

The main limitation of this study is its retrospective and single-center design. On the other hand, all kidney transplant candidates’ results were assessed by one nephrologist (AK) in the same manner, which diminished the potential inter-researcher bias. There is also a lack of data concerning past or current smoking habits among the study subjects; moreover, there are no follow-up data available, whereas data concerning the timing of statin/fibrate use are not precisely highlighted. We also have no data concerning cardiovascular outcomes post-transplantation.

## 5. Conclusions

The present study may suggest that even in asymptomatic kidney transplant candidates with normal results of primary cardiac screening tests (i.e., electrocardiogram and echocardiography), further deepening of cardiac evaluation should be considered, if other routinely available imaging methods reveal strong evidence of diffuse atherosclerosis. Such an approach may shorten the time of overall pre-transplant checkup and expedite active listing. In addition, there is an urgent need for well-designed clinical studies to address this issue in the majority of asymptomatic kidney transplant candidates.

## Figures and Tables

**Table 1:** Clinical characteristics of the study cohort at the time of the pre-transplant evaluation.

Parameter	N = 346
Age [years]	48.4 (47.0–49.9) Range, 18–80
Sex [M/F]	217/129
BMI [kg/m^2^]	25.8 (25.2–26.4)
Dialysis vintage * [months]	14.5 (6–33) Range, 0–166
Dialysis modality [n, %] HD CAPD Previous CAPD/current HD	253 (73.1) 26 (7.6) 25 (7.2)
Preemptive [n, %]	42 (12.1)
Residual diuresis * [mL]	1000 (500–1500)
Primary renal disease [n, %] Glomerulonephritis Diabetic nephropathy Pyelonephritis ADPKD Hypertensive nephropathy Other Unknown	98 (28.3) 31 (9.0) 15 (4.3) 53 (15.3) 32 (9.2) 69 (19.9) 48 (14.0)
Transplant No. (1/2/3)	292/46/8
Hypertensive [n, %]	329 (95.1)
Diabetics [n, %]	64 (18.5)
Ischemic heart disease [n, %]	42 (12.1)
Myocardial infarct or stroke [n, %]	24 (6.9)
Previous cardiac stenting [n, %]	18 (5.2)
CABG [n, %]	5 (1.4)

* Medians with Q1-Q3 values, or frequencies.

## Data Availability

The data presented in this study are available on request from the corresponding author due to privacy and ethical restrictions.

## Abbreviations

The following abbreviations are used in this manuscript:

ADPKD	Autosomal dominant polycystic kidney disease
BMI	Body mass index
CABG	Coronary artery bypass grafting
CAPD	Continuous ambulatory peritoneal dialysis
CKD	Chronic kidney disease
CTA	Coronary computed tomography angiography
ECG	Electrocardiography
HD	Hemodialysis
IHD	Ischemic heart disease
PET	Positron emission tomography
SPECT	Myocardial perfusion scintigraphy by single-photon emission CT
